# Breast Milk Lipidome Is Associated With Maternal Diet and Infants' Growth

**DOI:** 10.3389/fnut.2022.854786

**Published:** 2022-07-06

**Authors:** Joaquim Calvo-Lerma, Marta Selma-Royo, David Hervas, Baoru Yang, Linda Intonen, Sonia González, Cecilia Martínez-Costa, Kaisa M. Linderborg, Maria Carmen Collado

**Affiliations:** ^1^Institute of Agrochemistry and Food Technology (IATA-CSIC), Valencia, Spain; ^2^Department of Applied Statistics and Operations Research, and Quality, Universitat Politècnica de València, Valencia, Spain; ^3^Department of Life Technologies, Food Sciences, University of Turku, Turku, Finland; ^4^Department of Functional Biology, University of Oviedo, Oviedo, Spain; ^5^Diet, Microbiota and Health Group, Instituto de Investigación Sanitaria del Principado de Asturias (ISPA), Oviedo, Spain; ^6^Pediatric Gastroenterology and Nutrition Section, Hospital Clínico Universitario Valencia, INCLIVA Research Center, Valencia, Spain

**Keywords:** breast milk composition, fatty acids profile, lactation, diet, dietary habits, polyunsaturated fatty acids

## Abstract

**Objectives:**

The fatty acid (FA) composition of breast milk is a relevant aspect related to the development of the lactating infant. The present study aimed at exploring correlations between dietary intake of macro- and micronutrients with the FA profile in breast milk, and the possible implication for infants' growth.

**Study Design:**

Breast milk samples from a cohort of lactating women were collected 7–15 days postpartum. The FA profiles in triacylglycerol (TAG) and phospholipid (PL)-rich fractions were analyzed by gas chromatography. Diet was registered during the third trimester of pregnancy by means of a food frequency questionnaire (FFQ). In addition, anthropometric measurements of infants were collected from gestation and up to 12 months postpartum.

**Results:**

The FA profile in breast milk was characterized by a median of 37.4, 41.3 and 16.8% of saturated, monounsaturated, and polyunsaturated FAs, respectively. From the dietary components, zinc, iron, and B group vitamins were correlated positively with the proportion of total n-3 FAs in TAG and C20:5 n-3 in PL. Lycopene, vitamin E, zinc, and vitamin B2 showed a similar correlation with total polyunsaturated fatty acid (PUFA), total n-6 FAs, C20:4 n-6, and C18:2 n-6 in TAG. Regarding food groups, nuts showed the strongest association with several PUFA both in TAG and PL, while the vegetable group was also positively associated with C18:3 n-3. Furthermore, the concentration of linolenic acid (C18:3 n-3) and palmitic acid (C16:0) were positively associated with increased length for age (LFA) and weight for age (WFA) at 12 months compared with birth [ΔLFA −0.16 (−0.85, 0.37); ΔWFA −0.26 (−0.77, 0.21)].

**Conclusions:**

Mothers' intake of nuts, dietary sources of zinc, iron, and B group vitamins were identified as potential predictors of a high-unsaturated FA profile in breast milk. In addition, linolenic and palmitic acids in breast milk were positively associated with infants' growth in the first year of life.

## Introduction

Breast milk is a complex fluid containing nutrients, bioactive compounds, metabolites, immunoglobulins, and microorganisms, overall conforming to the most suitable source of feed for the infants. Such are the health benefits that the WHO recommends exclusive breastfeeding up to the age of 6 months, and then, after combining it with complementary feeding, until the age of 2 years, or beyond (1).

The composition of breast milk is known to depend on the mothers' characteristics, and several studies have addressed the implications of the maternal environment on the possible changes. In this sense, the maternal diet has proved to be one of the major determinants of the lipid composition of milk (2), and the microbiota profile (3). However, few studies address the contribution of food groups and dietary components to the lipid composition of breast milk (4).

Among the constituents of breast milk, the fat fraction is known to be one of the most important variable depending on the lactation stage and the moment of the day. Its content during the first month is ~3–4 g/dl, and some series report inter-subject variations, from 1.8 to 8.9 g/dl (5, 6). Fat is not only a source of energy but also a supply of essential FAs that are crucial for normal development, such as normal weight and height gain during lactation (7). In this regard, FAs deserve special attention, as supported by several relations to the prevention of developing allergic diseases (8) and intestinal development (9) or with the potential in brain development attributed to some of the polyunsaturated fatty acids (PUFA) (10). So, assessing the relationship between the composition of FAs in breast milk and the infant's growth is another worthwhile purpose.

Therefore, the present study aimed to perform a multivariable analysis to identify food groups and dietary components correlating with the FA composition of breast milk samples. Secondarily, the other objective was to assess the possible implication of certain FAs in breast milk on infants' growth.

## Materials and Methods

### Study Population

**Figure 1:**
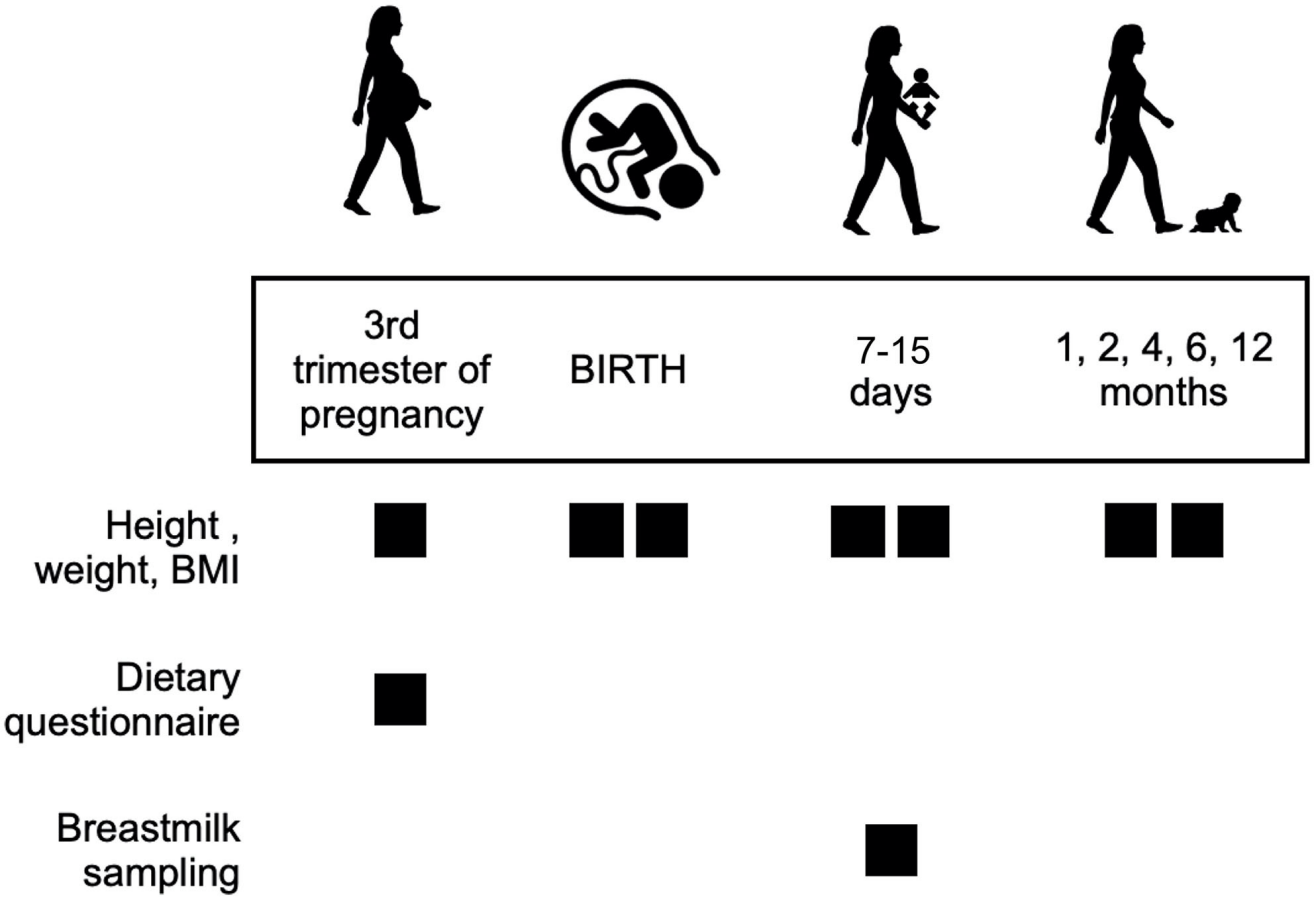
Overview of the study design.

This study is part of the MAMI cohort (11), a prospective longitudinal birth cohort with biological samples and clinical, anthropometrical, and dietary records performed in the Mediterranean area (València, Spain) The protocol was approved by the Hospital Ethics Committees (Hospital Clínico Universitario de Valencia, Valencia, Spain). The study was registered on the ClinicalTrial.gov platform (NCT03552939) and performed according to the World Medical Association's Declaration of Helsinki. Families were informed about the characteristics and duration of the study based on the patients' information sheet, and an informed consent form was signed. Figure 1 provides a summary of the study design.

### Breast Milk Samples Collection

In this study, the available breast milk samples were collected during the first 7–15 days after birth, with available records on the maternal dietary questionnaire and infant anthropometric data completed. These samples were included according to a standardized procedure in the instructions sheet that was previously described (3). Briefly, the mothers collected the milk samples by the use of a sterile pumper in the morning to standardize the collection among participants, avoiding collection from a breast that had been used for nursing within the past 2–3 h (5). The mothers brought the samples to the research center, or the samples were collected from their homes on the day of sampling. All the samples were frozen and stored at −70°C until the FAs were analyzed.

### FA Profile Analysis in Breast Milk

The FA compositions of neutral lipid (TAG-rich fraction, hereafter TAGs) and polar lipid (PL-rich fraction, hereafter PLs) fractions were analyzed from each sample in duplicate. Samples were thawed at room temperature and gently mixed, after which the internal standard mixture consisted of triheptadecanoin (Larodan Fine Chemicals AB, Malmö, Sweden) and dinonadecanoylphosphatidylcholine (Larodan Fine Chemicals AB, Malmö, Sweden) was added to the sample. The total lipids were extracted from samples with a modified Folch procedure (12) using chloroform, methanol, and 0.88% potassium chloride in water in a two-phase extraction. Extracted lipids were separated into TAG-rich and PL-rich fractions by solid-phase chromatography using silica cartridges (Waters, Dublin, Ireland) (12). The TAG-rich fraction was eluted from the column with dry diethyl ether (Merck KGaA, Germany) and the PL-rich fraction with methanol (Honeywell, Riedel-de Haen, France). FA methyl esters were prepared from the isolated fractions with sodium-methoxide-catalyzed transesterification. The methyl esters were analyzed with the Shimadzu GC-2010 with AOC-20i auto-injector and flame ionization detector and GC solution software (Shimadzu Corp., Kyoto, Japan). A splitless/split injection with split opened after 1 min was used. A wall coated open tubular column DB-23 (60 m × 0.25 mm i.d., liquid film 0.25 μm, Agilent technologies, J.W. Scientific, Santa Clara, CA, United States) was used to separate the methyl esters. The following temperatures were used: injector 270°C; oven initial temperature 130°C, hold 1 min, rate 4.5°C/min to 170°C, hold 0 min, rate 10°C/min to 220°C, hold 14.5 min, rate 60°C/min to 230°C, hold 3 min; detector 280°C. Helium was used as a carrier gas and 68D (Nu-Check-Prep, Elysian, MN, United States), Supelco 37 Component FA methyl ester Mix (Supelco, St. Louis, MO, United States), and 11A (Nu-Check-Prep, Elysian, MN, United States) as external standards. Results were expressed as the molar percentage of each FA in its lipid category and as milligram per milliliter of milk. Additionally, the percentage of polar lipids (as phosphatidylcholines) from all lipids was calculated.

### Anthropometric and Dietary Data

Anthropometric measurements in the children (length and weight) were obtained from the medical records during regular maternity clinic visits longitudinally at birth and thereafter at 7 days, 1, 2, 4, 6 and 12 months. From anthropometric data, the body mass index (BMI) was calculated, and the *z*-scores of length for age (LFA), weight for age (WFA), and BMI for age were electronically computed using the WHO Anthro software (www.who.int/childgrowth/software/en/). The WHO Child Growth Standards provide child growth measures standardized by age and sex. Additionally, the change (Δ = delta or change) in the LFA, WFH, and BMI z-scores from birth to 12 months of infants' age were calculated (ΔLFA, ΔWFA, and ΔBMI).

The maternal data collected in the food frequency questionnaire (FFQ) was used to estimate food consumption, which was also transformed into energy and macronutrient intake using the food composition tables of *Centro de Enseñanza Superior de Nutrición Humana y Dietética* (CESNID) (13). Aminoacids and FAs series were estimated from the United States Department of Agriculture (USDA) food composition database (U.S. Department of Agriculture Agricultural Research Service, Food Data Central. Available online: fdc.nal.usda.gov). The polyphenols content in foods was completed using the Phenol Explorer database (14) and fiber components and were ascertained using the food composition tables by Marlett et al. (15). In addition, the consumption of different food groups was estimated and expressed as mean grams intake, such as milk and dairy products, meat, eggs, fish, vegetables, fruit, nuts, legumes, cereal, vegetal oil, butter and margarine, pastries, sugars and processed foods (Supplementary Table S1).

Additional information was recovered from the medical records of the participants (both mothers and infants) because of their possible implication on the study outcomes: maternal pregestational body weight, BMI, weight increased during pregnancy, and birth mode (vaginal or C-section), duration of lactation, or use of probiotic supplements.

### Statistical Analysis

Data were summarized using mean [standard deviation (SD)] and median (1st and 3rd quartiles) in the case of numerical variables and by relative and absolute frequencies in the case of categorical variables. Dietary patterns in the study cohort were previously defined by Jensen–Shannon distance and partitioning around medoid clustering (3). The association between the FA profile and the dietary intake was assessed by adjusting a Sparse Partial Least Squares model (sPLS) and depicted in figures (16). The relevant, non-zero associations according to the adjusted sPLS model, among the several FAs in the breast milk and macro- and micronutrients in the diet, were depicted in the figures.

Optimization of the sPLS hyperparameters was performed using cross-validation. The association between breast milk FA composition and infant growth was assessed using horseshoe-regularized Bayesian linear regression models (17). In addition, 95% credible intervals were calculated for all estimations from the models and the ROPE method was used to determine the significance of the results. These models included as co-variables maternal BMI, type of delivery (vaginal or C-section), type of feeding (exclusive breastfeeding or mixed feeding), and duration of lactation. All statistical analyses were performed using R (version 4.1.2) and R-packages mixOmics (version 6.16.3), clickR (version 0.8.1), and brms (version 2.16.1).

## Results

### Clinical Data

A total of 111 subjects from the initial sample gave breast milk samples. Of those, 85 completed nutritional assessment.

Clinical data are shown in Table 1. The mean gestational age was 39.4 (±1.2) weeks. The mean pre-gestational weight and BMI were 62.6 kg (±10.1) and 23.1 kg/m^2^ (±3.6), respectively, with a mean weight increase of 12.4 (±4.7) kg throughout gestation. Most of the subjects (58%) had a vaginal delivery, and 49.3% of the newborns were female. The mean duration of lactation was 8.4 (±4.1) months, being exclusive during 4.1 (±2.2) months in 75.5% of the sample.

**Table 1:** Characteristics of the study subjects having breast milk sample to assess lipidome (*n* = 111).

**Gestational age (weeks)**	**39.4 (±1.2)**
Pre-gestational body weight (kg)	62.6 (±10.1)
Pre-gestational BMI (kg/m^2^)	23.1 (±3.6)
Weight increase (kg)	12.4 (±4.7)
**Type of delivery**	
Vaginal	58%
C-section	42%
**Newborn gender**	
Male	50.7%
Female	49.3%
**Type of feeding**	
Exclusive breast feeding	75.5%
Mixed feeding	24.5%
Duration of lactation (months)	8.4 (±4.1)
Duration of exclusive breast feeding (months)	4.1 (±2.2)

At birth, the median weight and length of the infants were 3.32 (3.02, 3.59) kg [LFA z-score 0.06 (−0.47, 0.99)] and 50.00 cm (48.5, 51.0) [WFA z-score 0.04 (−0.54, 0.68)] (Table 2). At the end of the follow-up period, the median z-scores for LFA and WFA were, respectively, 0.13 (−0.5, 0.59) and 0.00 (−0.78, 0.77), this representing, with respect to birth, ΔLFA and ΔWFA of −0.32 (−1.18, 0.48) and −0.02 (−0.55, 0.57).

**Table 2:** Anthropometric measurements of infants (*n* = 111) of the study cohort from birth to 12 months of follow-up, expressed as median values and 1st and 3rd quartiles.

	**Birth**	**7 days**	**1 month**	**2 months**	**4 months**	**6 months**	**12 months**
Weight (kg)	3.32 (3.02, 3.59)	3.30 (3, 3.6)	4.1 (3.8, 4.5)	5.12 (4.75, 5.55)	6.52 (6.03, 7)	7.45 (6.97, 8)	9.4 (8.68, 10.1)
WFA z-score	0.04 (−0.5, 0.68)	−0.33 (−0.83, 0.29)	−0.36 (−0.89, 0.22)	−0.24 (−0.85, 0.23)	−0.26 (−0.69, 0.23)	−0.23 (−0.69, 0.3)	0.13 (−0.5, 0.59)
Length (cm)	50 (48.5, 51)	51 (49.4, 52)	54 (52.5, 56)	57.5 (56, 59)	62.5 (61, 64.5)	66.5 (65, 68.3)	75 (72.5, 77)
LFA z-score	0.06 (−0.47, 0.99)	0.04 (−0.76, 0.67)	0.14 (−0.86, 0.91)	−0.03 (−0.96, 0.71)	−0.19 (−0.73, 0.65)	0.16 (−0.72, 0.68)	0.0 (−0.78, 0.77)
BMI (cm/m^2^)	13.19 (12.6, 14.2)	12.8 (12, 13.65)	13.9 (13.3, 15.1)	15.62 (14.7, 16.3)	16.52 (15.8, 17.4)	16.8 (16.1, 17.7)	16.9 (16, 17.9)
BMI z-score	−0.15 (−0.65, 0.6)	−0.34 (−1.06, 0.22)	−0.52 (−1.06, 0.21)	−0.33 (−0.78, 0.21)	−0.20 (−0.82, 0.19)	−0.22 (−0.69, 0.4)	0.22 (−0.45, 0.91)
ΔWFA z-score	–	−0.11 (−0.57, 0.46)	−0.41 (−0.7, −0.01)	−0.4 (−0.71, 0)	−0.27 (−0.81, 0.13)	−0.27 (−0.83, 0.28)	−0.26 (−0.77, 0.21)
ΔLFA z-score	–	−0.42 (−0.7, 0.06)	−0.34 (−0.86, 0.24)	−0.25 (−0.81, 0.29)	−0.31 (−1.02, 0.39)	−0.26 (−0.81, 0.35)	−0.16 (−0.85, 0.37)
ΔBMI z-score	–	−0.22 (−0.73, 0.11)	−0.34 (−0.87, 0.31)	−0.38 (−0.92, 0.31)	−0.23 (−1.01, 0.38)	−0.15 (−0.91, 0.55)	−0.1 (−0.94, 0.66)

### FAs Profile in Breast Milk

The breast milk samples at <15 days after birth (mean 11.25 ± 4.01 days) were characterized by a median TAG composition of 29.3 mg/ml (17.7, 39.4) and PL composition of 0.44 mg/ml (0.33, 0.63). The median composition of the three types of FAs in TAG was the following: 37.4% (35.7, 42.6) saturated (SFA), 43.1% (39.2, 46.1) monounsaturated (MUFA), and 18.3% (15.5, 20.1) polyunsaturated (PUFA) (Table 3). Regarding the total n-3 and n-6 FAs, the median values were 5.88 and 16.7%, respectively. The principal FA in TAG was oleic acid (C18:1 n-9), representing 39% of the fat fraction of breast milk, followed by palmitic acid (C16:0) at 21.2% and linoleic acid (C18:2) at 15.2%. In PL, however, the saturated C16:0 and C18:0 were the most abundant, noting that the polyunsaturated linoleic (C18:2 n-6) was also present in a relevant proportion.

**Table 3:** Fatty acid (FA) composition in triacylglycerol and phospholipid rich-fractions in median (1st Q and 3rd Q) quantification (mg/ml) and weight % (in italics).

	**Fatty acid composition in triacylglycerols (TAG) (mg/mL, *weight %*)**	**Fatty acid composition in phospholipids (PL) (mg/mL, *%*)**
C12:0	1.44 (1.07, 1.98)	0 (0, 0.01)
	*5.03 (3.97, 5.78)*	*0.91 (0.63, 1.19)*
C14:0	1.64 (1.24, 2.09)	0.01 (0.01, 0.02)
	*5.26 (4.52, 6.42)*	*2.73, (1.89, 3.89)*
C16:0	6.77 (4.53, 8.62)	0.08 (0.06, 0.13)
	*21.2 (19.2, 22.2)*	*21 (18.7, 27)*
C18:0	1.81 (1.35, 2.37)	0.08 (0.06, 0.1)
	*5.58 (5.11, 6.11)*	*21.4 (18.3, 22.4)*
C20:0	0.06 (0.04, 0.08)	0 (0, 0)
	*0.18 (0.16, 0.2)*	*0.31 (0.25, 0.36)*
C22:0	0.01 (0, 0.02)	0 (0, 0)
	*0.05 (0, 0.07)*	*0 (0, 0.06)*
**Total SFA**	**12.4 (8.99, 15.2)**	**0.2 (0.15, 0.27)**
	***37.4 (35.7, 42.6)***	***53.2 (49.8, 56.2)***
C14:1 (*n-*5)	0.04 (0.02, 0.06)	0 (0, 0)
	*0.11 (0.08, 0.15)*	*0.1 (0, 0.15)*
C16:1 (*n-*7)	0.42 (0.26, 0.59)	0 (0, 0)
	*1.34 (1.07, 1.64)*	*0.39 (0.31, 0.52)*
C18:1 (*n-*9)	12.4 (8.49, 16.0)	0.06 (0.04, 0.08)
	*39.5, (35.7, 41.5)*	*15.6 (14.3, 17.5)*
C18:1 (*n-*7)	0.51 (0.34, 0.63)	0 (0, 0)
	*1.46 (1.38, 1.62)*	*0.91 (0.82, 0.97)*
C20:1 (*n-*9)	0.16 (0.10, 0.20)	0 (0, 0)
	*0.48 (0.44, 0.52)*	*0.9 (0.77, 1.07)*
C22:1 (*n-*9)	0.04 (0.02, 0.05)	0 (0, 0)
	*0.11 (0.1, 0.13)*	*0.19 (0.15, 0.22)*
C24:1 (*n-*9)	0.02 (0.02, 0.03)	0 (0, 0)
	*0.08 (0.06, 0.1)*	*0 (0, 0)*
**Total MUFA**	**13.6 (9.2, 17.7)**	**0.07 (0.05, 0.09)**
	***43.1 (39.2, 46.1)***	***18 (16.9, 20.1)***
C18:2 (*n-*6)	5.35 (3.04, 6.71)	0.07 (0.05, 0.09)
	*15.2 (12.5, 17.1)*	*17.7 (16.2, 19.5)*
C18:3 (*n-*3)	0.17 (0.12, 0.28)	0 (0, 0)
	*0.55 (0.42, 0.7)*	*0.19 (0.15, 0.29)*
C20:2 (*n-*6)	0.17 (0.11, 0.22)	0 (0, 0)
	*0.47 (0.41, 0.56)*	*0.63 (0.52, 0.72)*
C20:3 (*n-*6)	0.11 (0.00, 0.20)	0.01 (0, 0.01)
	*0.42 (0, 0.55)*	*1.72 (1.34, 2.03)*
C20:4 (*n-*6)	0.15 (0.09, 0.25)	0.02 (0.01, 0.03)
	*0.5 (0.41, 0.66)*	*4.66 (3.76, 5.6)*
C20:3 (*n-*3)	0.02 (0.01, 0.02)	0 (0, 0)
	*0.05 (0.03, 0.05)*	*0.18 (0, 0.26)*
C20:5 (*n-*3)	0.02 (0.01, 0.02)	0 (0, 0)
	*0.06 (0.04, 0.08)*	*0.11 (0, 0.19)*
C22:6 (*n-*3)	0.17 (0.11, 0.22)	0.01 (0.01, 0.01)
	*0.49 (0.39, 0.65)*	*2.44 (2.01, 3.17)*
**Total PUFA**	**6.32 (3.77, 7.67)**	**0.12 (0.08, 0.14)**
	***18.3 (15.5, 20.1)***	***28.5 (16, 31.2)***
Total *n-*3	0.39 (0.29, 0.56)	0.01 (0.01, 0.02)
	*1.11 (0.99, 1.54)*	*2.82 (2.51, 3.61)*
Total *n-*6	5.88 (3.44, 7.32)	0.1 (0.07, 0.13)
	*16.7 (14.2, 18.6)*	*25 (23.1, 27.6)*
*n-*3/ *n-*6 ratio	*0.07 (0.07, 0.08)*	*0.11 (0.11, 0.13)*

*SFA, saturated fatty acids; MUFA, monounsaturated fatty acids; PUFA, polyunsaturated fatty acids*.

### Is the Breast Milk FA Profile Associated With Maternal Dietary Intake?

#### Maternal Dietary Patterns: Food and Nutrient Intakes

The dietary intake of the subjects was studied because of the possible implications for the breast milk composition. According to the nutrient intake, the dietary intake could be previously characterized by two patterns: diets with high-animal protein and high-fat, and diets with high plant-based protein and high-fiber (3). Referring to these patterns in terms of food groups, the first cluster, compared with the second one, was defined by a higher intake of meat (median 170 vs. 96 g/day) and sugars (104 vs. 0 g/day) and a lower intake of fruits (229 vs. 500 g/day), vegetables (422 vs. 573 g/day), and cereals (109 vs. 165 g/day) (Table 4).

**Table 4:** Characterization of the maternal diets (*n* = 85) during pregnancy expressed in food groups and nutrients (mean g/day), according to the two clusters previously defined in the study cohort (3).

**Cluster**	**1**	**2**
	***n* = 37**	***n* = 48**
Milk and dairy	288.8 (182.1, 421.4)	344.5 (103.6, 391.9)
Cereal	165.7 (139.3, 205.7)	109.6 (90.0, 154.1)
Vegetal oils	40 (40, 50)	40 (40, 41.4)
Butter and margarine	0.8 (0.0, 5.1)	0.8 (0.0, 3.4)
Fruit	500.2 (381.4, 612.1)	229.3 (163.2, 317.1)
Vegetables	537.5 (422.3, 666.1)	422.1 (252.8, 477.3)
Legumes	51.4 (31.4, 74.3)	51.4 (30.0, 62.9)
Nuts	11.3 (4.0, 21.4)	6.3 (2.0, 17.1)
Fish	81.2 (52.9, 104.3)	94.8 (54.8, 128.6)
Meat	96.0 (74.0, 129.3)	170.7 (125.0, 230.7)
Sugar	0.0 (0.0, 10.0)	104.0 (50.3, 200.8)
Pastries	98.3 (60.0, 150.0)	78.7 (48.8, 141.6)
Processed foods	37.1 (16.7, 71.5)	42.0 (17.6, 111.4)

Despite dietary habits in terms of food groups were defined in four clusters, the statistical analysis showed no evidence on a significant association between the clusters and the FA profile in breast milk samples, or the infants' growth within the study period.

#### FAs Profile and Maternal Nutrients and Food Groups Intake

**Figure 2:**
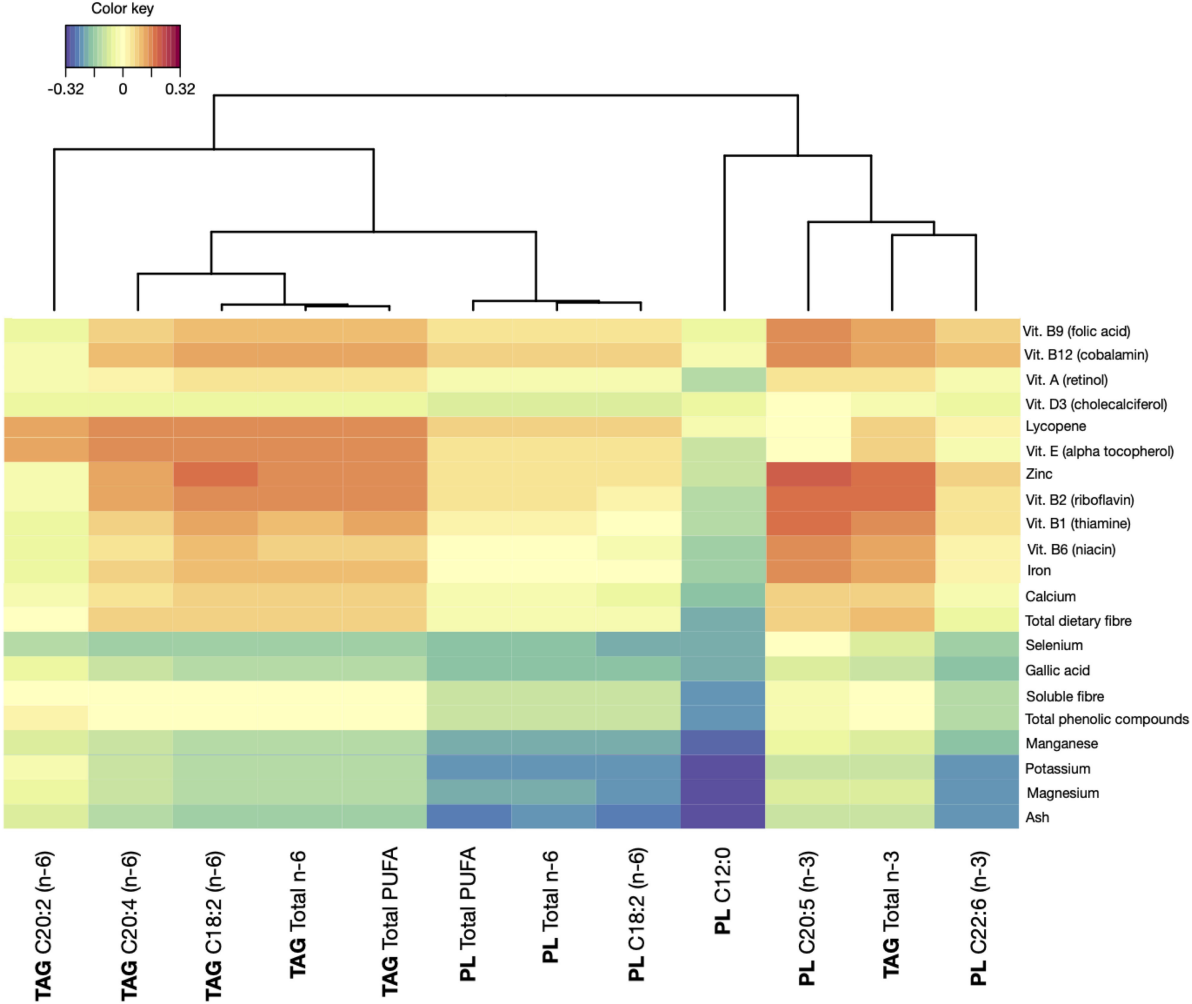
Correlations between dietary compounds and levels of fatty acids (FAs) series in breast milk samples. TAG, triacylglycerol molecules; PL, phospholipid molecules.

The FA profile of breast milk samples was associated with specific maternal dietary compounds consumed in a regular diet in the 85 subjects having both a dietary intake registry and a breast milk sample to assess lipidome. The significant associations between dietary components and FAs in breast milk samples, considering both TAG and PL, are presented in Figure 2.

The group of n-6 PUFA from TAG was associated the most with dietary intake. Concretely, a significant association was found between the intake of lycopene and vitamin E, both liposoluble compounds with antioxidant activity, and C20:2, C20:4 C18:2, total *n*-6 FAs, and total PUFA. In addition, zinc, vitamin B2, vitamin B1, and vitamin B12 showed a strong association with these compounds except for C20:2. Vitamin B9, vitamin B6, and iron also showed an association with C20:4, C18:2, total n-6 FAs, and total PUFA. Focusing on total n-3 FAs, zinc and vitamin B2 contributed to a significant increase. This was also observed, with lower correlations, for vitamin B6, iron, and total dietary fiber. Apart from the total n-3 FAs, only C20:5 n-3 in phospholipids showed a positive dependence with the dietary intake, concretely with vitamin A, vitamin D, vitamin B9, vitamin B12, zinc, vitamin B2, B1, B6, and iron. On the other hand, some FAs in the breast milk samples showed a decreasing correlation with dietary compounds. This was the case of C12:0 in PL, for which the intake of vitamin B3, potassium, magnesium, manganese, phenolic compounds, and soluble fiber contributed to lower presence. Similarly, niacin, potassium, and magnesium were inversely related to the presence of C22:6 n-3 in phospholipids in breast milk. These same compounds showed to be inversely related with total PUFA in phospholipids, total n-6 FAs in phospholipids, and C18:2 n*-*6 in phospholipids. In addition, the presence of niacin seemed to have a negative impact on the presence of other compounds, such as total PUFA in triglycerides, total n-6 FAs in triglycerides, and C18:2 and C20:4 in TAG. It is worthy of noting that none of the macronutrients showed significant associations with FA composition of the breast milk samples.

**Figure 3:**
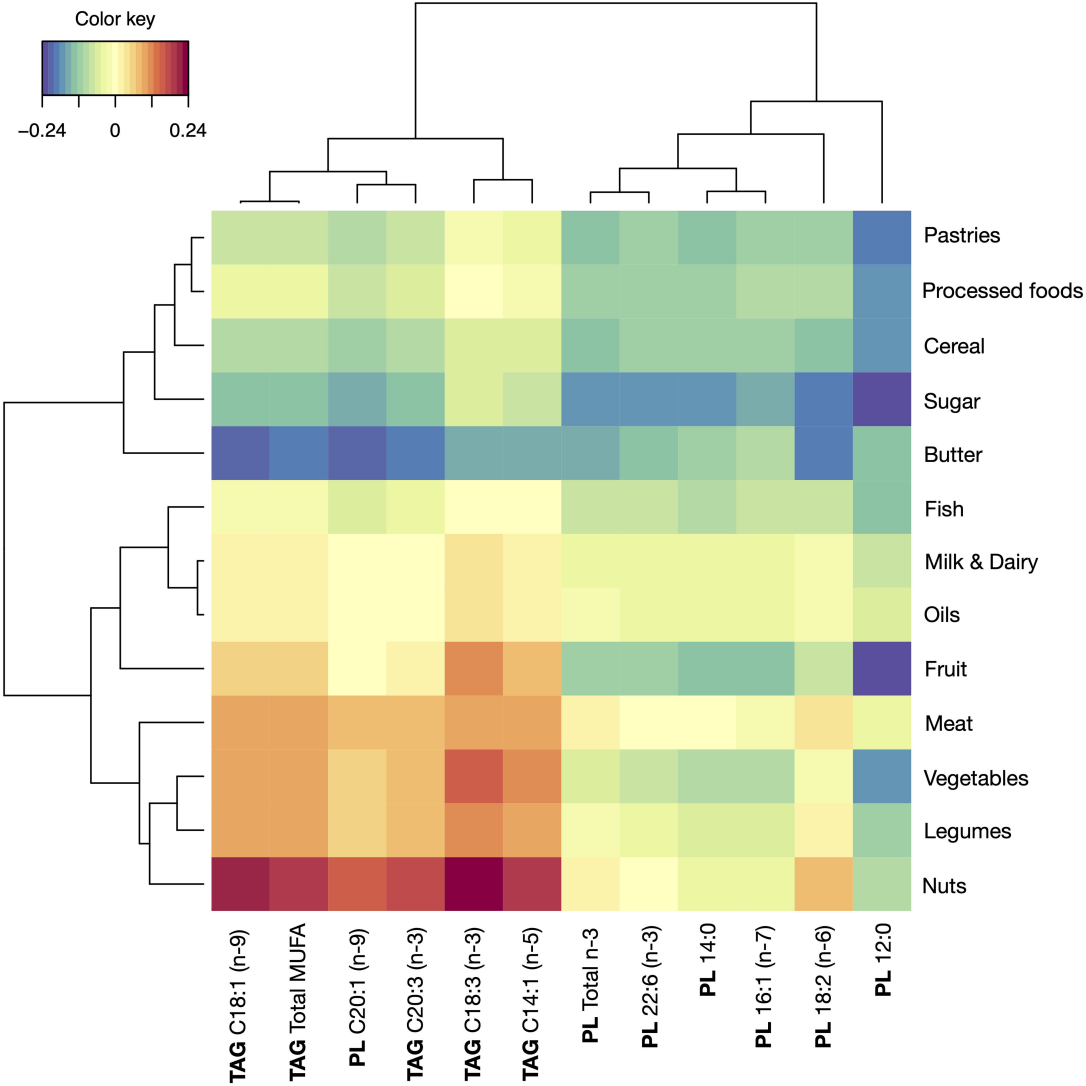
Correlations between foods and levels of FAs series in breast milk samples. TAG, triacylglycerol molecules; PL, phospholipid molecules.

From a different perspective, the food groups conforming the diet of the subjects were assessed also against the FA composition of the breast milk samples (Figure 3). According to the results, nuts intake was correlated with increased total MUFA in TAG, from which C18:1 (oleic acid) was also identified as increased. However, the highest correlation was found for C18:3 n-3 (alpha-linolenic), which is an n-3 PUFA abundant in many vegetable oils, seafood and walnuts. Additionally, C14:1 (myristoleic) and C20:3 n-6 (dihomo-γ-linolenic) also showed a positive correlation with nuts intake. A similar pattern as nuts was found in the intake of meat, legumes and vegetables, and a contrasting inverse relationship between these FAs with butter intake. Another relevant observation related to the positive correlation between legumes intake and two relevant n-3 FAs, C18:3 (also detected in vegetables intake), and C20:3 n*-*3. As for the group conformed by FAs in PL (C12:0, C14:0, C16:1, C18:2, C22:6, and total n-3 FAs) a cluster of food groups, most of them considered as non-recommendable, showed a negative correlation: sugar, pastries, processed foods, butter, and cereals.

### Is the Breast Milk FAs Profile Associated With Infants' Growth?

**Figure 4:**
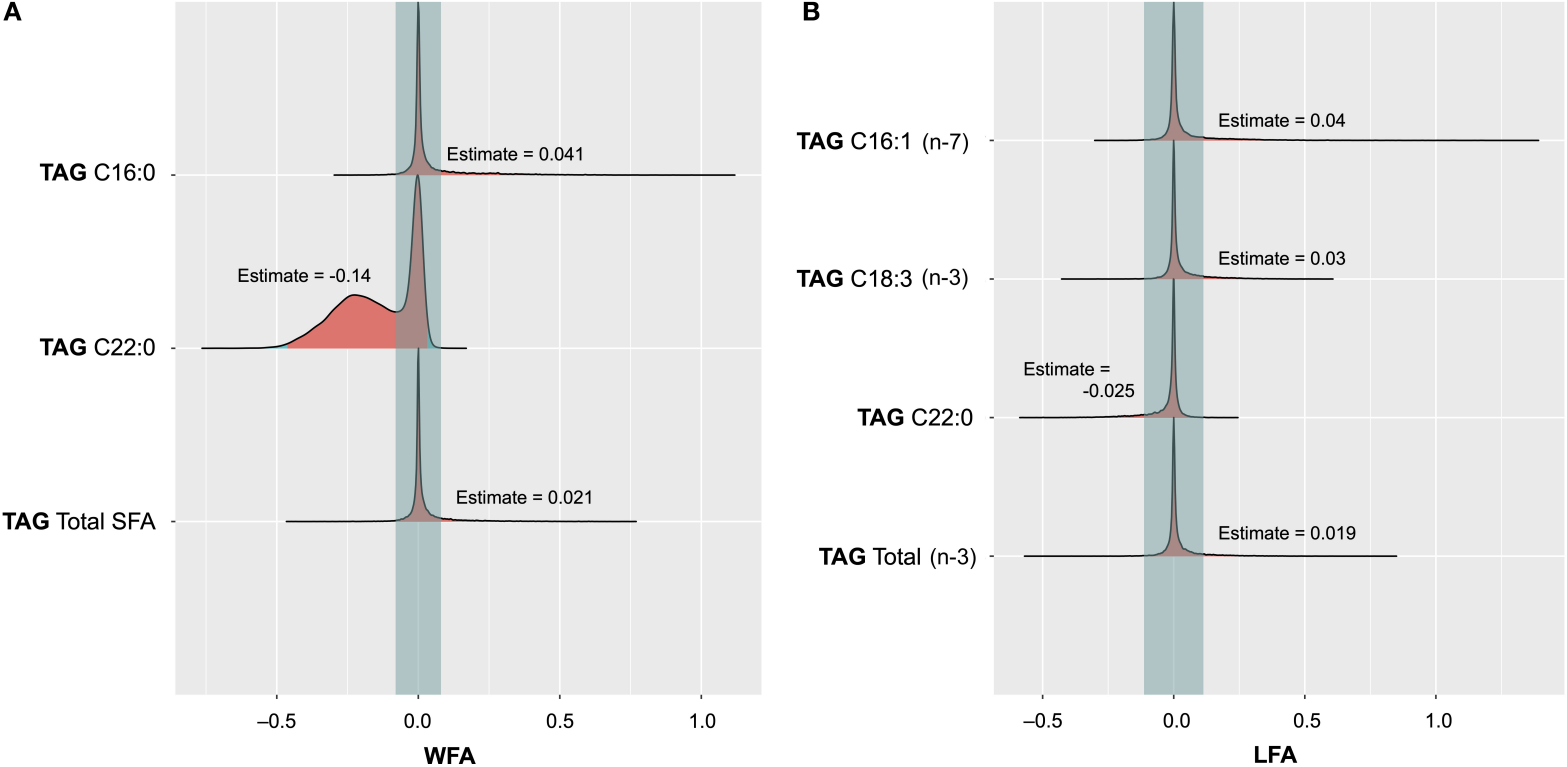
Posterior distribution plots for the selected variables depicting the effect of FAs in triacylglycerols (TAG) in breast milk on the change of length for age (LFA) *z*-score **(A)** and weight for age (WFA) *z*-score **(B)** at the end of the follow-up period (12 months) with respect to birth.

Related to the secondary objective of this study, associations between breast milk lipidome and infants' growth in terms of time-related change along a 12-month period in LFA and WFA z-scores were explored in a total of 111 milk-infant pairs (Figure 4). Focusing on the growth, two FAs in TAG, concretely palmitoleic (C16:1) and linolenic (C18:3 n-3), were shown to be positively associated, with LFA. The estimated values indicate an increase of 0.04 and 0.03, respectively, in the LFA z-score at 12 months per each mg/ml of the FA. Regarding the change in WFA, palmitic acid (C16:0) was positively associated, and also, total SFA showed a similar positive association, estimate values being 0.041 and 0.021, respectively. In contrast, behenic acid (C22:0) was inversely associated with both LFA and WFA, in terms of decreased z-score values of −0.025 and −0.14, respectively, per each mg/ml of this FA. In terms of maternal and perinatal characteristics, type of delivery, type of feeding, or duration of lactation had no show significant effects on change in LFA or WFA.

## Discussion

This study addressed the possible associations between relevant parameters related to maternal-infant health during the first months of life: FA composition in breast milk, infants' growth, and maternal diet. The findings highlight the implication of the maternal diet on the FA profile in breast milk samples, and also include the association of some of the FAs in breast milk with infants' growth.

Focusing first on the FA profile in the breast milk samples, the results were in accordance with previous literature. Mäkelä et al. (18) reported in a cohort of normal-weight woman (as in the present one) from Finland a proportion of 43, 40, and 14% of saturated, monounsaturated, and PUFA, respectively, while in ours the percentage of total PUFA was slightly higher at the expense of the saturated. This discrepancy could be related to the different dietary habits among the studied countries (18). In fact, geographical locations are known to impact the breast milk composition of FAs in breast mild, and concretely, differences in n-3 and n-6 FAs between Spanish and Finnish breast milk samples have been reported before (19).

The main finding relates to the identification of the dietary compounds having a significant impact on breast milk composition in FAs. In this sense, zinc consumption was shown to be one of the major determinants of the content of n-6 PUFAs in TAG, and also contributed to the amount of total n-3 FAs. Among the observed associations of zinc, the one with arachidonic acid (C20:4 n*-*6) was previously reported in breast milk samples (20). The authors attributed this relationship to zinc being a relevant co-factor for essential FAs metabolism. A similar finding was found in plasma and cells (21, 22). In addition, the deficiency of zinc in different contexts was shown to have an association with reduced essential FAs in serum samples (23, 24). Therefore, a similar rationale could be attributed to the other observed associations between zinc and C20:2, C18:2, total n-6 FAs, and total PUFA. Another relevant association was found between total n-3 FAs and vitamins from the B group. In particular, the association with vitamin B12 might have relevant implications in the brain function development of the lactating infants, but the mechanisms by which this vitamin may interact with n-3 FAs remain unknown (25). In contrast, no studies have been found in the literature specifically addressing the possible relationship between minerals, such as potassium, magnesium, and manganese in the diet and the lipid profile in human milk, which in the present study showed a negative effect on n-6 and n-3 FAs in PL. However, it should be considered that most of the dietary sources of these minerals come along with PUFA. Considering the dietary intake effect in terms of food groups, nuts had the highest degree of association in the most relevant FAs in TAG, such as oleic, linolenic and dihomo-γ-linolenic (C20:3 n-6) acids, and total MUFA. This finding could be explained by the high content of unsaturated FAs in nuts. Along with nuts, the vegetables group showed a high degree of association with linolenic acid, possibly because of the presence of green vegetables, which contain this FA (26), although the amount is low compared with other dietary sources. Addressing both results together, it can be concluded that diets high in nuts, zinc, and vitamins from the B group are associated with a more unsaturated FA profile in breast milk. A relevant remark at this point relates to the different associations of dietary compounds with the FAs in TAG than in PL, and the fact that the latter are only in minor proportion while TAG represent 98% of the lipid content of breast milk (9).

Finally, a relevant result relates to the identification of the FAs in breast milk that are associated with lactating infants' growth over a period of 12 months. In fact, this question is so relevant that the formulation of infant formula tries to adapt the composition of FAs to enhance growth (27, 28). In the present study, diverse types of FAs were identified as relevant for LFA and WFA. Concretely, saturated palmitic acid (C16:0), one of the most abundant FAs in breast milk and the major saturated fat, was associated with WFA at 12 months. This result is consistent with previous literature, showing that high dietary intake of saturated fat induces body weight gain (29, 30), but no references specifically addressing this relation in the context of lactation were found. On the other hand, PUFA like alpha-linolenic acid (C18:3), an n-3 FA that is present in relatively low amounts in breast milk, showed evidence of contributing to increased growth in terms of LFA. While different studies have addressed the presence of this FA as a relevant factor related to growth (31), linoleic acid content in breast milk in two geographical population was not associated with different patterns of infants' growth in a previous study (32). Nonetheless, our finding is in accordance with a previous birth cohort study, showing that, among 26 assessed FAs in breast milk, linolenic acid was associated with the length-for-age *z*-score at 52 and 104 weeks (33). In addition, a relevant finding is the significant effect in reduced LFA and WFA attributed to behenic acid (C22:0). This FA is present in very low amounts in breast milk (median value 0.001 mg/ml), not being detected in some of the assessed samples. Further studies in breast milk composition should take into account behenic acid, as at the moment, none of the available literature details any specific role of this FA.

The present study is not exempt of some limitations: diet was only collected in the third trimester of pregnancy, and dietary intake relies on a study subject's self-reported data. Nutrient composition databases do not fully match the exact food reported, and also, there are uncertainties with extrapolating frequency data to exact amounts. The best approach would have included a food record at the breast milk collection time. In addition, to better assess the correlation between infants' growth and the FA composition of breast milk, we should have evaluated possible complementary dietary sources in the infants. On the other hand, a systematic error is inherent to the FFQ method to estimate dietary intake, leading to overreporting for some of the assessed compounds (34). However, this error does not prevent from comparing individuals within the same cohort. On the other hand, the study samples are considered as transitional milk (<15 days from birth), so the content of fat would differ from the other stages, such as colostrum or mature milk.

The results encourage further research considering a more in-depth analysis of dietary intake coinciding with the breast milk sampling timepoint and including a registry of infants' complementary feeding to assess the effect of breastfeeding on growth in a longer follow-up period, so that the contribution of specific FAs in milk to growth could be confirmed and further explained. Ideally, interventional studies addressing the effect of the identified dietary factors on breast milk composition in FAs should be undertaken.

## Conclusions

In conclusion, the dietary factors that contribute to a high-unsaturated FA profile in breast milk are identified, such as the intake of nuts, and sources of zinc and vitamins from the B group. Additionally, palmitic, palmitoleic, and alpha-linolenic acids in breast milk should be closely monitored in future studies because of their association with infants' growth.

## Data Availability Statement

The raw data supporting the conclusions of this article will be made available by the authors, without undue reservation.

## Ethics Statement

This study was reviewed and approved by the Ethics Committees of the hospitals involved in MAMI Project (Hospital Universitario y Politécnico La Fe, Hospital Clinico Universitario de Valencia, as well as by the Local Ethics Committee of Atención Primaria-Generalitat Valenciana (CEIC-APCV). The study is registered on the ClinicalTrial.gov platform, with the registration number NCT03552939. Written informed consent to participate in this study was provided by the participants. Written informed consent to participate in this study was provided by the participants' legal guardian/next of kin.

## Author Contributions

KL and MC conceived and designed the study. MC, CM-C, and KL supervised the project development. JC-L, MS-R, DH, SG, and LI conducted the research and participated in data curation. JC-L, MS-R, SG, and KL were involved in the methodology and data management. DH and JC-L performed the statistical analysis. MC and CM-C participated in funding acquisition. KL and BY provided resources and support for FA composition analysis. JC-L wrote the first draft of the manuscript. All authors contributed to manuscript revision, read, and approved the submitted version.

## Funding

This study was supported through a 5-year grant from the European Research Council (ERC)—European Union's Horizon 2020 Framework—with an ERC Starting Grant (Ref. 639226).

## Conflict of Interest

The authors declare that the research was conducted in the absence of any commercial or financial relationships that could be construed as a potential conflict of interest. The handling editor AG-I declared a shared affiliation with the authors JC-L, MS-R, and MC at the time of review.

## Publisher's Note

All claims expressed in this article are solely those of the authors and do not necessarily represent those of their affiliated organizations, or those of the publisher, the editors and the reviewers. Any product that may be evaluated in this article, or claim that may be made by its manufacturer, is not guaranteed or endorsed by the publisher.
