# Supplementary material for: Breast Milk Lipidome Is Associated With Maternal Diet and Infants' Growth
Source: Front Nutr. 2022 Jul 6;9:854786. doi: 10.3389/fnut.2022.854786 (PMC9296781; doi:10.3389/fnut.2022.854786)
Supplement: Supplementary file 1 [file Table_1.docx]

**Supplementary table S1.** Food items included in the food groups

| Milk & dairy | Whole-fat milk, semi-skimmed milk, skimmed milk, yoghurt, skimmed yoghurt, fresh cheese, mild cheese, cured cheese. |
| --- | --- |
| Cereal | White bread, whole-grain bread, pasta, whole-grain pasta, rice, whole-grain rice |
| Vegetal oils | Olive oil, sunflower oil, corn oil |
| Butter | Butter, margarine |
| Fruit | Orange, mandarin, banana, apple, pear, strawberry, cherry, peach, watermelon, melon, kiwi, grapes |
| Vegetables | Spinach, broccoli, lettuce, tomato, carrot, pepper, asparagus, artichoke, onion, green bean, potato, mushrooms |
| Legumes | Lentil, chickpea, beans |
| Nuts | Walnuts, almonds, peanuts |
| Fish | Salmon, tuna, hake, seafood |
| Meat | Chicken, turkey, pork, lamb, beef, ham |
| Processed meat | Pate, black pudding, coldmeats, minced meat, sausages, hamburger |
| Sugar | Table sugar, honey, soft drinks, jam |
| Pastries | Croissant, puffy pastry, biscuits, donut, breakfast cereal |
| Processed foods | Dairy desserts, ready-made foods, industrial pizzas, industrial breaded fish/meat |
